# Prediction model for day 3 embryo implantation potential based on metabolites in spent embryo culture medium

**DOI:** 10.1186/s12884-023-05666-7

**Published:** 2023-06-08

**Authors:** Rong Liang, Sheng Nan Duan, Min Fu, Yu Nan Chen, Ping Wang, Yuan Fan, Shihui Meng, Xi Chen, Cheng Shi

**Affiliations:** 1 Reproductive Medical Center, Department of Obstetrics and Gynecology, Peking University People’s Hospital, Peking University, Beijing, China; 2 Beijing National Laboratory for Molecular Sciences (BNLMS), MOE Key Laboratory of Bioorganic Chemistry and Molecular Engineering, College of Chemistry and Molecular Engineering, Peking University, Beijing, China

**Keywords:** Metabolomics, Spent embryo culture medium, Human day 3 embryos, Implantation prediction, LC-MS

## Abstract

**Background:**

Metabolites in spent embryo culture medium correlate with the embryo’s viability. However, there is no widely accepted method using metabolite dada to predict successful implantation. We sought to combine metabolomic profiling of spent embryo culture medium and clinical variables to create an implantation prediction model as an adjunct to morphological screening of day 3 embryos.

**Methods:**

This investigation was a prospective, nested case-control study. Forty-two day 3 embryos from 34 patients were transferred, and the spent embryo culture medium was collected. Twenty-two embryos implanted successfully, and the others failed. Metabolites in the medium relevant to implantation were detected and measured by Liquid Chromatography-Mass Spectrometry. Clinical signatures relevant to embryo implantation were subjected to univariate analysis to select candidates for a prediction model. Multivariate logistical regression of the clinical and metabolomic candidates was used to construct a prediction model for embryo implantation potential.

**Results:**

The levels of 13 metabolites were significantly different between the successful and failed groups, among which five were most relevant and interpretable selected by Least Absolute Shrinkage and Selection Operator regression analysis. None of the clinical variables significantly affected day 3 embryo implantation. The most relevant and interpretable set of metabolites was used to construct a prediction model for day 3 embryo implantation potential with an accuracy of 0.88.

**Conclusions:**

Day 3 embryos’implantation potential could be noninvasively predicted by the spent embryo culture medium’s metabolites measured by LC-MS. This approach may become a useful adjunct to morphological evaluation of day 3 embryos.

**Supplementary Information:**

The online version contains supplementary material available at 10.1186/s12884-023-05666-7.

## Background

In vitro fertilization embryo transfer (IVF-ET) is currently the most effective treatment for infertility, and millions of couples in China are treated with this technology every year. However, improvement of live birth rate is still relatively limited [1]. To increase the success rate, clinicians often transfer two or more embryos, which, in turn, increases the occurrence of multiple births and obstetric complications and ultimately does little to improve the live birth rate [2]. Thus, the best way to improve the live birth rate of IVF-ET is selecting the single embryo with the highest implantation potential.

Morphological evaluation is routinely used in IVF-ET laboratories. However, morphology alone cannot accurately screen embryos with high implantation potential because of the subjective nature of the screening criteria [3]. Preimplantation genetic screening (PGS), which uses chromosomal diploid as a criterion, has been developed recently. Although PGS is more objective, testing requires the removal of 4–5 cells from the embryo; thus, PGS is invasive with respect to the embryo [4, 5]. In addition, chimerism often exists in early embryos, so the chromosomes of the removed cells do not always match those of the remaining embryo, which makes the results of PGS uncertain [6]. Thus, PGS is not widely used for embryo screening in China.

In IVF laboratories, embryos are cultured in microdroplets made of culture medium. Embryos absorb the contents of the culture fluid and metabolize small molecules. Thus, changes in the metabolic substances in the microdroplets after culturing can reflect the viability of the embryos, which provides objective and noninvasive methods for embryo evaluation. Several assays [7–9] have been used to find metabolites associated with embryo implantation potential, but due to differences in assay methods, sample size, sample collection, and statistical analysis methods, no metabolomic assay has been reported for clinical use worldwide [10]. Clearly, more accurate noninvasive implantation prediction models are needed that are based on metabolite analysis.

As one of the most advanced and powerful methods of metabolite profiling, Liquid Chromatography-Mass Spectrometry (LC-MS) is widely used in omics research. However, LC-MS is rarely used for human embryo culture medium analysis. Eldarov et al. [11] used LC-MS to report day 5 culture medium metabolomic profiles; they found significant differences between embryos with different morphological classes, between euploid and aneuploid embryos in the same class, and between embryos with successful and unsuccessful implantation. However, the investigators did not devise any prediction model for embryo implantation potential based on these metabolite differences.

In this study, we collected spent culture medium of day 3 embryos from IVF-ET patients, detected metabolites in the medium by LC-MS, and searched for metabolites that had levels correlated with successful and failed implantation outcome. In addition, we sought clinical variables that were correlated with implantation outcome. Lastly, we combined the correlated metabolites and clinical variables to develop a model to predict the implantation potential of day 3 embryos, with a view to using the model as an adjunct to morphological assessment of embryo selection.

## Materials and methods

### Study design and participants

This investigation was a prospective, nested case-control study. The patients who intended to undergo IVF-ET and day 3 embryo transfer were included. Exclusion criteria were as follows: patients who got no oocyte or only degenerated oocytes after controlled ovarian hyperstimulation and oocytes retrieval; patients who did not have normally fertilized zygotes (with two pronuclei 16–18 h after insemination); patients with endometrial dysplasia (endometrial thickness less than 6 mm at the implantation window or no typical “trilinear sign”), endometriosis, hydrosalpinx, adenomyosis or uterine malformation and patients with single gestational sac after double embryo transfer. This study was approved by the Ethics Review Board of the Peking University People’s Hospital (2018PHB061-01). Each participant was informed in detail about the study’s procedures and risks, and the consent was obtained. The research was conducted in accordance with the World Medical Association’s Declaration of Helsinki.

### Ovulation stimulation, oocyte retrieval, embryo culture, transfer, and follow-up

A controlled ovulation treatment such as long, ultra-long, and microstimulation protocol was performed for each patient according to their ovarian function. Thirty-six hours after human chorionic gonadotropin (hCG) administration, transvaginal ultrasound-guided oocyte retrieval was performed. Forty hours after hCG administration, insemination was performed by conventional in vitro fertilization (IVF) or intracytoplasmic sperm injection (ICSI). For IVF, the retrieved oocyte corona cumulus complexes were incubation with sperm for 16-18 h before the pronuclei was assessed. The cumulus and the corona of the cells were removed by a set of pipettes with consecutive inner diameters of 220, 150 and 140 μm. For ICSI, oocytes were enzymatically denuded by means of brief exposure to 75IU/mL hyaluronidase (SAGE) and then mechanically denuded by a set of pipettes as mentioned above. During the process of pronuclei assessment, the embryos were denuded if there were residual granulosa cells. During the denuding process, the embryos were extensively washed in culture medium before culture. Sixteen to eighteen hours after insemination, pronucleus formation was observed. Normally fertilized (two pronuclei) embryos were individually distributed in 40 ul of pre-equilibrated cleavage medium (Cook, American) in a triple gas incubator (5% CO_2_ + 6% O_2_ + 89% N_2_) until the third day. Morphological evaluation of day 3 embryos was performed using the modified Pruissant scoring criteria [12]. Day 3 embryos were classified into four grades according to the speed of oocyte cleavage, uniformity of blastomeres, and fragmentation ratio. Embryos with grade I or II were selected for transfer or freezing. Patients for whom all embryos were frozen received thawing embryo transfer treatment. Blood level of hCG was measured 14 days after transfer to determine the clinical pregnancy. Transvaginal ultrasound was conducted 28 days after transfer to determine the number of gestational sac.

### Spent culture medium sample collection and grouping

Within two hours after the embryos were processed (transferred or frozen), 25 ul of spent embryo culture medium was removed from the microdroplets of each embryo, placed in small brown glass vials, marked well, and stored at -80 degrees. The samples were grouped according to the follow-up results. If the patient became pregnant after transfer and the number of gestational sacs visible by ultrasound was the same as that of transferred embryos, which indicated successful implantation, the corresponding spent culture medium samples were classified as the successful implantation group (s-implanting group). If the patient did not become pregnant, the corresponding culture medium samples were classified as the failed implantation group (f-implanting group).

### LC-MS metabolomic profiling of spent embryo culture medium

Spent embryo culture medium samples for LC-MS testing were prepared by mixing 20 uL aliquots of medium with 100 uL methanol and 2 uL 500 nM ^13^ C-serine which served as internal standard. The mixture was centrifuged at 14,000 rpm, 4 ℃ for 30 min to precipitate proteins, and 100 uL supernatant was collected, dried under vacuum, and reconstituted in 35 uL MeOH/H_2_O (1:1, v/v).

Analysis of all metabolites was performed on an LC-MS system consisting of a Thermo Scientific Q Exactive MS with an ESI source (Thermo, USA) and a Thermo Scientific Dionex Ultimate 3000 HPLC (Thermo, USA). Data acquisition and processing were performed using Excalibur (Thermo, USA). The HPLC separation was performed on an Xbridge Amide column (2.1 mm × 100 mm, 3.5 μm, Waters, USA) at 20℃. 10 mM ammonium acetate, pH 9.0 (solvent A) and acetonitrile (solvent B) were employed as the mobile phase. A gradient was used consisting of 85 − 42% B for 5 min, 42 − 5% B for 11 min, 5% B for 8 min, 5–85% B for 1 min, and 85% B for 7 min. The flow rate of the mobile phase was 0.3 mL/min. For each run, the injection volume was 20 µL. The MS detection was performed under negative ESI mode. The metabolites were monitored using the full-scan mode within m/z range of 60–800. The AGC target and maximum inject time were 1e6 and 100. For source parameters, the sheath gas, aux gas, sweep gas flow rate, spray voltage, capillary temperature S-lens RF level and aux gas heater temperature were 35, 10, 2, 3.2, 350, 55 and 300, respectively. During this process of LC-MS testing, 13 C-serine was added to every sample and served as internal standard. Using Python 3.7, we divided the intensity values of each sample by the corresponding peak areas of internal standard reported by Xcalibur 2.2 (Thermo, USA).

For identification of target compounds, MS/MS was used in parallel reaction mode. The MS2 resolution, AGC target, maximum IT, and isolation window were 17,500, 2e5, 100 ms and 4.0 m/z. 20 and 40 NCE were simultaneously introduced to produce MS2 spectra.

### Statistical analysis

Continuous clinical variables were presented as mean ± standard and analyzed by unpaired t-test between the s- and f-implanting groups. Categorical clinical variables were expressed as frequency and percent and analyzed by Chi square test between the two groups. Multivariable logistic regression analysis based on a generalized estimate equation (GEE) model was employed to test the effect of each clinical variable on the implantation outcome. Only factors that showed significant effects were selected to construct the prediction model.

Metabolomic data of spent culture medium acquired from LC-MS (raw format) were first converted to ms1 format, which contains numerical intensity values. Using Python 3.7, we divided the intensity values of each sample by the corresponding peak areas of internal standard reported by Xcalibur 2.2 (Thermo, USA) and rewritten into the ms1 files. Finally, ms1 files were converted to standard mass spectrometry file mzML format. All format conversions were performed with msConvert (ProteoWizard [13]). The differential analysis was performed by PAIRWISE at XCMS online [14]. The MS2 spectra were analyzed by METLIN [15] to identify metabolites with different levels between the s-implanting and f-implanting group. Finally, Least Absolute Shrinkage and Selection Operator (LASSO) regression analysis was constructed to select the most relevant and interpretable set of metabolomic predictors from the metabolites with different levels. Multivariable logistic regression analyses were used to construct a noninvasive prediction model for day 3 embryo implantation potential. The model combined the clinical variables selected by univariate analysis and the most relevant and interpretable set of metabolomic predictors identified by LASSO regression. The Area Under Curve (AUC) of the model was calculated.

## Results

### Baseline characteristics of patients and embryos

**Fig. 1:**
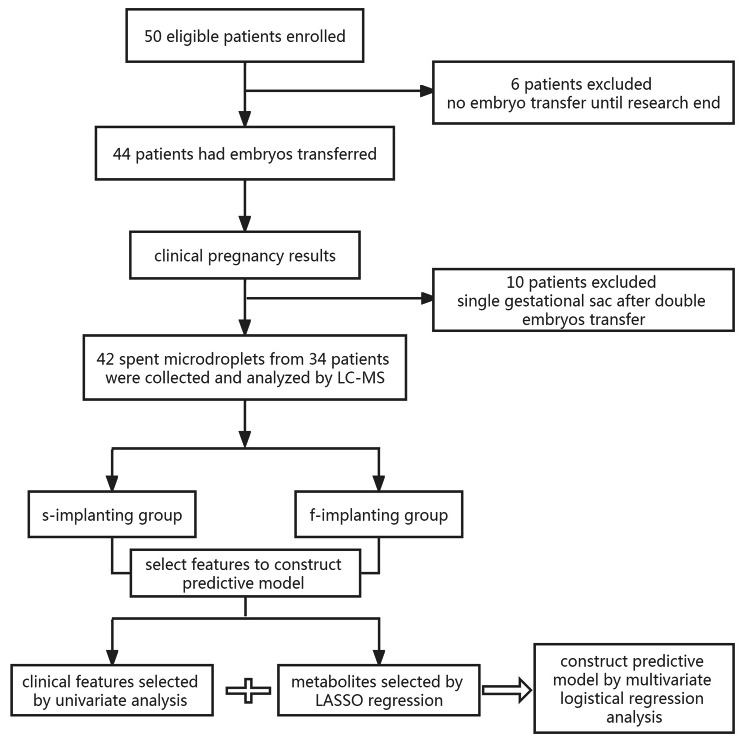
Flow chart of study design and analysis

Thirty-four patients were enrolled, and their 42 embryos were transferred (8 patients received two embryos, and 26 patients received a single embryo). The spent embryo culture medium of the 42 embryos was collected and analyzed by LC-MS. Eighteen patients (22 embryos) became pregnant after embryo transfer, whereas the other 16 patients (20 embryos) failed (Fig. 1). Table 1 lists the demographic and clinical characteristics of the two groups. There were no significant differences between the two groups in mean age of the women and their husbands, the woman’s body mass index, number of antral follicles, infertility duration, ovary stimulation duration, number of retrieved oocytes, and the thickness of the endometrium before transfer. Approximately 95.45%, and 90% transferred day 3 embryos were cleaved to 7–10 blastomeres in the s-implanting and f-implanting groups. Approximately 54.54% and 40% transferred embryos in the s-implanting and f-implanting groups were evaluated as morphologically graded I embryos. Approximately 68.18% and 45% transferred embryos in the s-implanting and f-implanting groups were inseminated by conventional IVF. Approximately 90.91% and 85% embryos in the s-implanting and f-implanting groups were transferred in fresh cycle. None of the above proportions between the two groups had significant differences.

**Table 1:** Demographic and clinical characteristics of patients who underwent day 3 embryo transfer and collection of spent embryo culture medium

	s-implanting (n = 18)	f-implanting (n = 16)	P value
Maternal age (years)	32.78 ± 3.89	34.31 ± 3.95	0.55
Paternal age (years)	34.50 ± 4.05	35.31 ± 4.11	0.76
Maternal BMI (kg/m^2^) ^a^	23.08 ± 4.21	22.50 ± 4.47	0.98
Infertility duration (years)	3.28 ± 2.12	2.12 ± 1.64	0.06
Ovary stimulation duration (days)	10.17 ± 3.26	9.56 ± 2.34	0.23
Antral follicles (numbers)	11.33 ± 5.59	9.19 ± 5.43	0.78
Retrieved oocytes (numbers)	7.72 ± 3.74	9.56 ± 4.20	0.16
Endometrial thickness ^b^ (mm)	10.17 ± 2.07	10.49 ± 2.87	0.16
Primary infertility, n (%)	12(66.67)	10(62.5)	0.80
Number embryos transferred	s-implanting (n = 22)	f-implanting (n = 20)	
embryos inseminated by IVF, n (%) ^c^	15(68.18)	9(45)	0.13
embryos transferred in fresh cycle, n (%)^d^	20(90.91)	17(85)	0.91
7–10 cell embryos, n (%) ^e^	21(95.45)	18(90)	0.60
morphologically graded I embryos, n (%) ^f^	12(54.54)	8(40)	0.37

^a^ BMI = body mass index

^b^ Endometrial thickness: the thickness of the endometrium on the LH surge day

^c^ Proportion of embryos inseminated by conventional in vitro fertilization

^d^ Proportion of embryos transferred in fresh cycle

^e^ Proportion of embryos with 7–10 blastomeres on day 3

^f^ Proportion of morphologically graded I embryos

### Effect of clinical variables on implantation of day 3 embryos

As shown in Table 2, older female participants had more embryos successfully implanted than younger participants (OR = 1.26, 95% CI: 0.84–1.90). In contrast, fewer embryos were successfully implanted in cases of older men participants, high body mass index, and long periods of infertility and stimulation of women participants (OR = 0.83, 95% CI: 0.54–1.27; OR = 0.97, 95% CI: 0.78–1.20; OR = 0.71, 95% CI: 0.48–1.07; OR = 0.94, 95% CI: 0.63–1.39). Compared with patients who had secondary infertility, fewer embryos were successfully implanted in patients diagnosed with primary infertility (OR = 0.60, 95% CI: 0.091–3.91). Compared with embryos that did not have 7–10 blastomeres and were not morphologically graded I embryos, embryos with 7–10 blastomeres and morphologically graded I embryos had equal implantation ability (OR = 1.00, 95% CI: 1.000–1.001; OR = 1.00, 95% CI: 0.999–1.000). However, based on P-value, none of the aforesaid clinical variables had a significant effect on implantation outcome.

**Table 2:** Multivariable logistic regression analysis based on a generalized estimate equation (GEE) model for implanted embryos *

	Implanted embryos ^a^	
Covariate	EXP(OR) 95%CI	P-value
Maternal age, year	1.26(0.84 ~ 1.90)	0.27
Paternal age, year	0.83(0.54 ~ 1.27)	0.39
Maternal BMI, kg/m^2^	0.97(0.78 ~ 1.20)	0.77
Infertility duration, day	0.71(0.48 ~ 1.07)	0.10
Type of infertility		
primary	0.60(0.091 ~ 3.91)	0.59
secondary	reference	
No. of antral follicles		
Length of stimulation, day	0.94(0.63 ~ 1.39)	0.75
No. of retrieved oocytes	0.93(0.76 ~ 1.13)	0.45
Type of cell for embryos		
7–10 blastomeres	1.00(1.000 ~ 1.001)	0.52
other	reference	
Type of quality for embryos		
morphologically graded I embryos	1.00(0.999 ~ 1.000)	0.26
other	reference	

^a^ whether the transferred embryo implanted successfully

* analysis performed by Generalized estimate equation model, subject ID = patient unique medical record number (independence)

### LC-MS and LASSO regression analysis

**Fig. 2:**
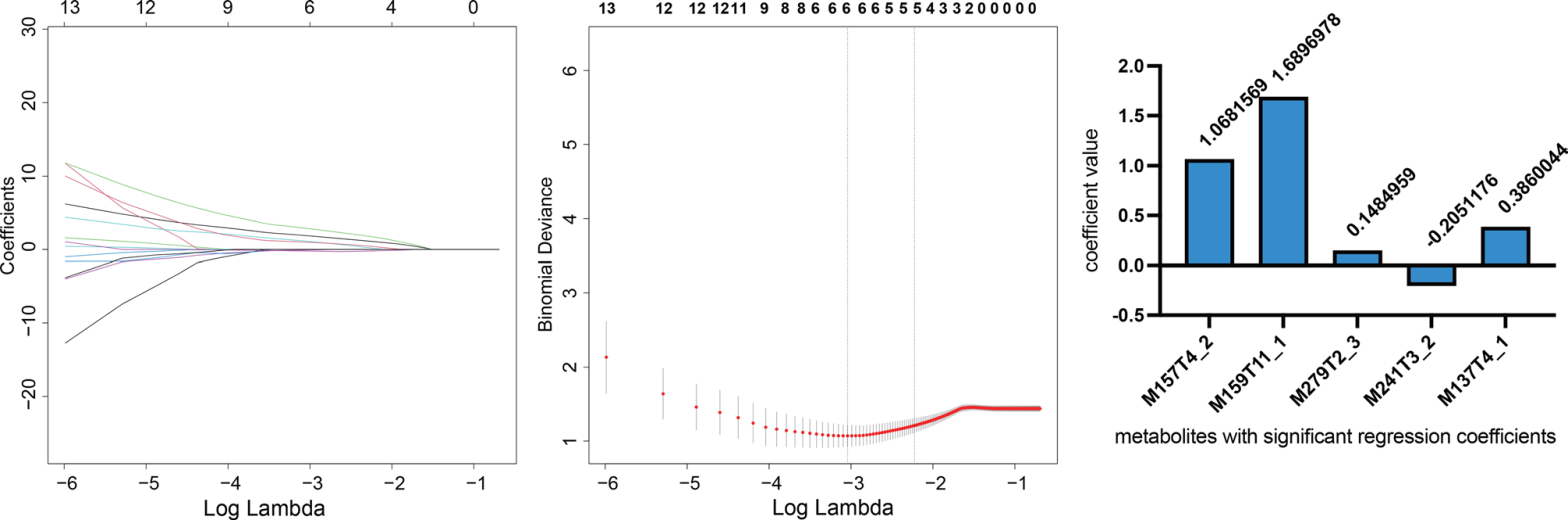
The process of selecting targeted metabolites by LASSO regression

The LC-MS analysis identified 13 metabolites that had a greater than one and one half-fold change in level when comparing the spent embryo culture medium from the s-implanting with the f-implanting group (p < 0.05). Nine metabolites had higher levels in the s-implanting group compared with the f-implanting group, and four metabolites were lower (Table 3). Five metabolites (M157T4_2, M159T11_1, M279T3_3, M241T3_2, and M137T4_1) had significant regression coefficients as assessed by LASSO (Fig. 2).

**Table 3:** Metabolites with different levels between s- and f-implanting groups spent embyro culture media

name	FC	P-value	mzmed	s-implanting(×10^7^)	f-implanting(×10^7^)
up-regulated ^a^ metabolites					
M157T4_2	2.318	0.001	157.1235	11.3 ± 7.6	4.9 ± 3.2
M187T3_1	1.291	0.023	187.0074	16.8 ± 5.1	13.0 ± 5.2
M159T11_1	1.751	0.003	158.9691	0.6 ± 0.3	0.3 ± 0.2
M279T3_3	1.458	0.024	279.2513	1.5 ± 0.7	1.1 ± 0.6
M144T4_1	1.493	0.041	144.0449	4.3 ± 2.4	2.9 ± 2.0
M137T4_1	1.507	0.035	137.0237	7.7 ± 4.1	5.1 ± 3.6
M239T9_3	2.737	0.030	239.0770	7.6 ± 9.6	2.8 ± 1.8
M175T8_1	6.833	0.031	175.0243	83.2 ± 143.7	12.2 ± 11.5
M176T8	6.413	0.034	176.0272	5.5 ± 9.6	0.9 ± 1.0
down -regulated ^b^ metabolites					
M249T8_2	4.538	0.014	249.0221	4.8 ± 10.7	21.7 ± 26.7
M241T3_2	2.193	0.015	241.1457	2.9 ± 2.9	6.4 ± 5.4
M298T3_1	2.578	0.022	298.1324	0.6 ± 0.7	1.5 ± 1.5
M197T3	2.249	0.027	197.1550	0.6 ± 0.8	1.4 ± 1.3

^a^ Compared with f-implanting group; the level of metabolites in the spent embryo culture medium of the s-implanting group were higher

^b^ Compared with f-implanting group; the level of metabolites in the spent embryo culture medium of the s-implanting group were lower

### Construction of prediction model based on metabolomic features

**Fig. 3:**
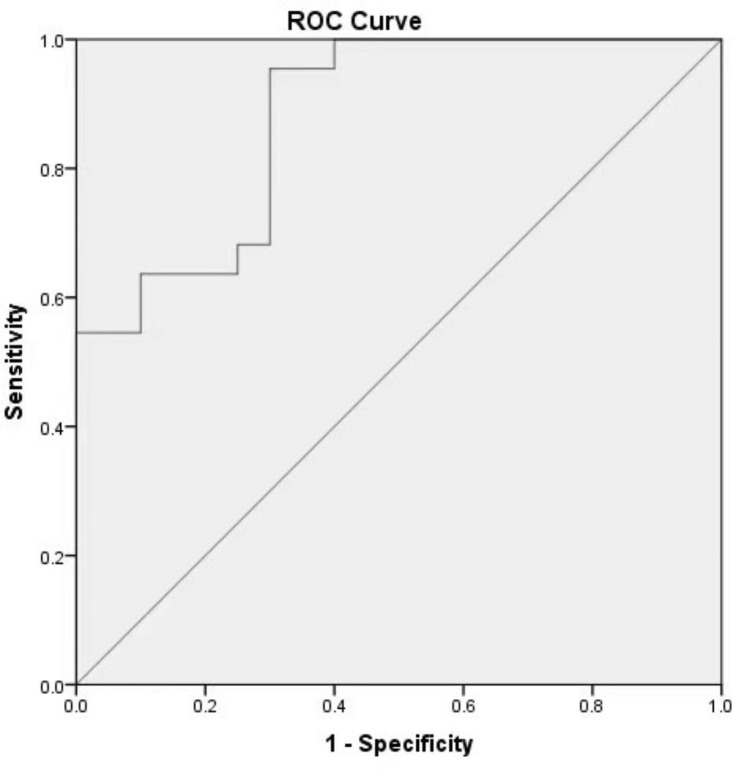
ROC curve for the prediction model by multivariable logistic regression analysis

Because no clinical feature had a significant correlation with embryo implantation potential, we used only metabolites identified by LASSO regression to construct the prediction model by multivariate logistical regression analysis. Embryos were more likely to implant successfully if the spent embyro culture medium had increased levels of M157T4_2, M159T11_1, M279T3_3, and M137T4_1 (OR = 26.949, 95% CI: 1.295-560.813, OR = 138.196, 95% CI: 1.741-10969.28, OR = 14.446, 95% CI: 0.562–371.05, OR = 4.639, 95% CI: 0.073-293.351). A high level of M241T3_2 in the medium had a negative effect on embryo implanting potential (OR = 0.486, 95% CI:0.151–1.563). According to the coefficient value from multivariate logistical regression analysis of these five metabolites, the prediction model formula was calculated as follows: Risk score = (3.294* M157T4_2) + (4.929* M159T11_1) + (2.670* M279T3_3) + (-0.721* M241T3_2) + (1.534* M137T4_1). The area under the ROC curve (AUC) of this model was 88% (Fig. 3).

## Discussion

Metabolites in the spent culture medium of an embryo provide information about the embryo’s developmental potential. Thus, it was desirable to detect the metabolites and construct a noninvasive evaluation method for selecting embryos with high implanting potential. However, there was no noninvasive metabolomic method in use worldwide to assist in embryo selection. Differences in study design contribute to this lack of a widely accepted metabolomic screening method. Therefore, additional study was needed to verify metabolite differences between embryos that achieve implantation and those that fail. Because day 3 embryos are transferred in our IVF center, we captured metabolite profiles of day 3 spent embryo culture medium to discover metabolite differences. We correlated these differences to predict implantation potential. Our model had an AUC of 0.88.

Whether implantation potential of day 3 embryos could be predicted by a spent embryo culture medium metabolomic approach has been investigated for several years. Some studies supported a spent embryo culture medium metabolomic approach. Zhao et al. used Raman spectroscopy to investigate metabolomic profiling of day 3 embryo culture media combined with morphology to successfully predict 6 out of 7 embryos implanting potential [16]. Bastu et al. who also used a Raman spectroscopy approach. Their model was reported to have a specificity and sensitivity of 80.25% and 87.50% by receiver operating characteristics (ROC) analysis [17]. Cortezzi et al. used electrospray ionization mass spectrometry to detect metabolites in embryo culture medium; they used partial least squares discriminant analysis to construct a prediction model. The model correctly identified 100% of samples from the implanted group and 70% of samples from the nonimplanted group [18]. Figoli et al. established a metabolomic-based approach and data modeling technique based on Fourier transform infrared spectroscopy to identify day 3 embryo’s culture medium (Vitrolife) fingerprints suitable and unsuitable for predicting implantation. Their model correctly identified only 60% of samples from the nonimplanted group, to which clinical variables contributed largely [19]. Seli et al. used near infrared spectroscopy to develope an embryo viability calculation based on metabolomic profiling of human embryo culture media; the correlation with reproductive potential of day 3 embryos was independent of morphology [20]. We also developed a noninvasive implantation prediction model with an accuracy of 88% based on LC-MS identification of metabolites and exclusion of clinical variables. Metabolomic technology, modeling technique, clinical variables, sample size, culture medium, and culture condition are all factors that affect metabolomic models for day 3 embryos. Thus, it is difficult to compare the accuracy of models and recommend one or more for routine clinical use. Moreover, there also some contrary opinions. Rinaudo et al. concluded that ^(1)^H NMR profiling of culture media cannot predict success of implantation for day 3 human embryos because they could not validate their borderline class separation established from set 1 of the media samples in the other set 4 samples [21]. Vergouw et al. reported that embryo selection by metabolomic profiling of spent embryo culture media with the use of near-infrared spectroscopy (NIR) spectroscopy plus morphology did not improve the live birth rate of day 3 embryos in a double-blind, randomized controlled trial [22]. In sum, we could form consensus only that spent embryo culture medium metabolomics may be robust approach to evaluate day 3 embryos. Additional randomized controlled trials with larger sample sizes are needed to assess the benefit of any metabolomic evaluation as an adjunct to conventional morphologic evaluation.

We used the METLIN database to identify the five metabolites that we used to build the prediction model. M137T4_1 was probably salicylic acid, M241T3_2 was probably 2-octylcyclopropane-1 or 1-dicarboxylic acid, and M279T3_3 was probably chaulmoograsauere or octadecadienoic acid; M157T4_2 and M159T11_1 were not identified in the database. Among the three identified molecular ions, only octadecadienoic acid (M279T3_3) is reported to be related to embryo development. Octadecadienoic acid is an unsaturated fatty acid, and a high ratio of octadecadienoic acid to other fatty acids in the culture medium has a significant positive effects on embryonic development in vitro [23]. Karaşahin found that high doses (1000 µM) of oleic acid (cis-9-octadecenoic acid or 9,12-octadecadienoic acid) in the culture medium increased the viability of bovine embryos after vitrification [24]. Aardema et al. also demonstrated that oleic acid had a positive effect on bovine oocyte developmental competence [23]. Nevertheless, there was no report of the effect of octadecadienoic acid on human embryo development and only limited study of the relationship between other unsaturated fatty acids and human embryo development. Haggarty et al. reported that preimplantation human embryos actively took up individual fatty acids at different rates at different stages of development, and there was little net accumulation before the 8-cell stage [25]. Our results indicate a positive relationship between the concentration of unsaturated fatty acids in microdroplets after embryo culture and embryo development, which was contradictory to the fact that pre-8-cell embryos rarely take up unsaturated fatty acids. Also using LC-MS technology, Eldarov described 15 decreased and 31 increased metabolites that differed between successful and failed implanted day 3 embryos [11]. As in our study, two molecular ions (m/z = 187.07 and m/z = 158.08) were present at similar levels in successful and failed implanted culture medium. The molecular ion with m/z = 187.07 was probably 2-phenylbutyric acid and the ion with m/z = 158.08 was unidentified. These two molecular ions would be useful for improving embryo culture medium.

Although our study provides a noninvasive prediction model for embryo implantation potential based on five metabolites, the study had limitations. There was no developmental outcome endpoint for the blastocysts; therefore, our model cannot provide suggestions for laboratories that cultivate all their zygotes to blastocysts. Also, our study had a small sample size, and the sensitivity and specificity of the model could not be assessed; therefore, additional samples are needed to verify the model.

## Conclusion

We developed a model that accurately predicted implantation potential of day 3 embryos that were morphologically graded I or II. The model was based on noninvasive LC-MS analysis of metabolites in the spent embryo culture medium. We suggest that the model will improve the accuracy of morphological embryo evaluation.

## Electronic supplementary material

Below is the link to the electronic supplementary material.

**Additional file 1**

## Data Availability

The datasets used or analyzed in this study are available from the corresponding authors on reasonable request.

## Abbreviations

IVF-ET: In vitro fertilization embryo transfer
PGS: Preimplantation genetic screening
LC-MS: Liquid Chromatography-Mass Spectrometry
hCG: human chorionic gonadotropin
ICSI: intracytoplasmic sperm injection
GEE: generalized estimate equation
LASSO: Least Absolute Shrinkage and Selection Operator
AUC: Area Under Curve
ROC: receiver operating characteristics
NIR: near-infrared spectroscopy.
